# Reprocessing seafood waste: challenge to develop aquatic clean meat from fish cells

**DOI:** 10.1038/s41538-021-00121-3

**Published:** 2022-01-27

**Authors:** Yusuke Tsuruwaka, Eriko Shimada

**Affiliations:** 1 Cellevolt, Niigata, Japan; 2 Institute for Advanced Biosciences, Keio University, Yamagata, Japan; 3 Marine Bioresource Exploration Research Team, Marine Biodiversity Research Program, Institute of Biogeosciences, Japan Agency for Marine-Earth Science and Technology (JAMSTEC), Kanagawa, Japan; 4 Division of Applied Biosciences, Graduate School of Agriculture, Kyoto University, Kyoto, Japan; 5 Department of Pharmacology, University of California, Davis, Davis, CA USA

**Keywords:** Cell biology, Sustainability

## Abstract

Fish consumption has been increasing worldwide as per capita consumption of fish rises along with population growth. At the same time, overfishing is increasing all over the world, causing enormous damage to the ecosystem. There is an urgent need to secure sustainable fishery resources to meet the expanding demand for fish. The present study focused on the cells obtained from fish fins, which were often discarded as food waste, and which had the potential to change their morphology with simple treatments, creating the possibility of processing fish fin cells into clean meat (i.e., meat produced in vitro; artificial, lab-cultured meat using tissue engineering techniques). The fin-derived fibroblast-like cells demonstrated an interesting characteristic; changing the sera or culture media supported differentiation of the fibroblast-like cells to various cell morphologies, such as neurofilaments and adipocytes, etc., without genetic manipulation. Furthermore, it was possible to culture the cells in multi-layered and three-dimensional forms that were suitable for processing and shaping. Taking advantage of the cells’ characteristics, ‘aquatic clean meat’ was produced successfully at the prototype stage. Our results suggest that fish fins, which are often treated as waste material, thus, are easy to procure, simple to process, and could be used to create a sustainable food resource.

## Introduction

In recent years, the technology of 3D cell culture has been developed and applied to a cell sheet^1^. Although these technologies have been applied in the medical field^2–4^, they are expected to be applied in other fields, such as the food industry^5,6^. Our aim is to explore applying this technology to produce marine food resources. As we currently face on the food crisis, population growth is resulting in a rapidly increasing demand for livestock products, which leads to environmental stress since raising livestock requires large amounts of natural resources and accounts for about 14.5% of total anthropogenic greenhouse gas emissions^7–9^. Therefore, the establishment of a sustainable food resource production system for the next generation will be beneficial for environmental and ecological protection^10,11^. In addition, a sustainable food production system is expected to reduce waste by recycling it effectively^12^.

Fish is increasingly consumed worldwide, resulting in enormous impacts on the ecosystem, including overfishing^13–15^. We have reported the farming of edible deep-sea fish that are difficult to rear, and their biological activity at the cellular level^16,17^. Fish farming needs to take into account its operating costs and rearing space. In the present study, we focused on the recycling of fish waste material and its possibility to develop ‘aquatic clean meat’ from fish cells to meet the challenge outlined in the United Nations’ (UN) Sustainable Development Goal 14: “Conserve and sustainably use the oceans, seas and marine resources for sustainable development”. We focused on fish fins because 1) they are often discarded as food waste and 2) collecting fresh fins partially does not unnecessarily take the lives of fish. If the technology were developed to create ‘aquatic clean meat’ from seafood waste, it would be an environmentally friendly way to increase animal welfare and sustainability. As a first step, we demonstrated the potential for and the advantages of obtaining cells from fins at the individual level here.

Fish have a high level of ability to regenerate. They are able to regenerate various body parts, including fins, which are equivalent to the hand and foot in humans, as well as hearts, neurons, and so on. In general, regeneration occurs by ‘dedifferentiation’ and/or self-renewal with basal stem cells^18–21^. Individual fish are able to regenerate their partially lost fins within a few weeks. Thus, fish can be a highly regenerative food material. In this study, we deal with partial fins cut from a fish’s body. Cultured fin tissues cut from fish bodies are known to show explants around the tissues in a culture flask^22,23^. Therefore, we investigated the cell variations that have experienced the regeneration process, including the differentiation potential if the cells have dedifferentiated.

In general, when embryonic stem (ES) cells (i.e., the inner cell mass of early embryos as well as amphibian undifferentiated embryonic cells) are cultured for differentiation induction in vitro, they are known to differentiate neural cells unless cultured with specific stimulating factors, that is, external signals such as serum, growth factors, etc. Thus, the basal state of undifferentiated cells was destined to neural induction intrinsically^24^. Shimada et al.^25^ reported that fins were derived from the somatic mesoderm which developed from somatic stem cells. This suggests the possibility that when fin cells in the regeneration process are dedifferentiated and become undifferentiated cells, they may become basal neural somatic stem cells. If dedifferentiated fish cells have the ability to differentiate to various types of cells, such as muscle, fat, fibers, and nerves, which are the main components of edible animal meat, then we can manipulate and arrange the proportion of these meat components to create ‘aquatic clean meat’. We obtained cells from the fins of waste materials and investigated their potential for cell differentiation, which could be applied to clean meat.

## Results

### Obtaining cell materials from fish fins

**Fig. 1:**
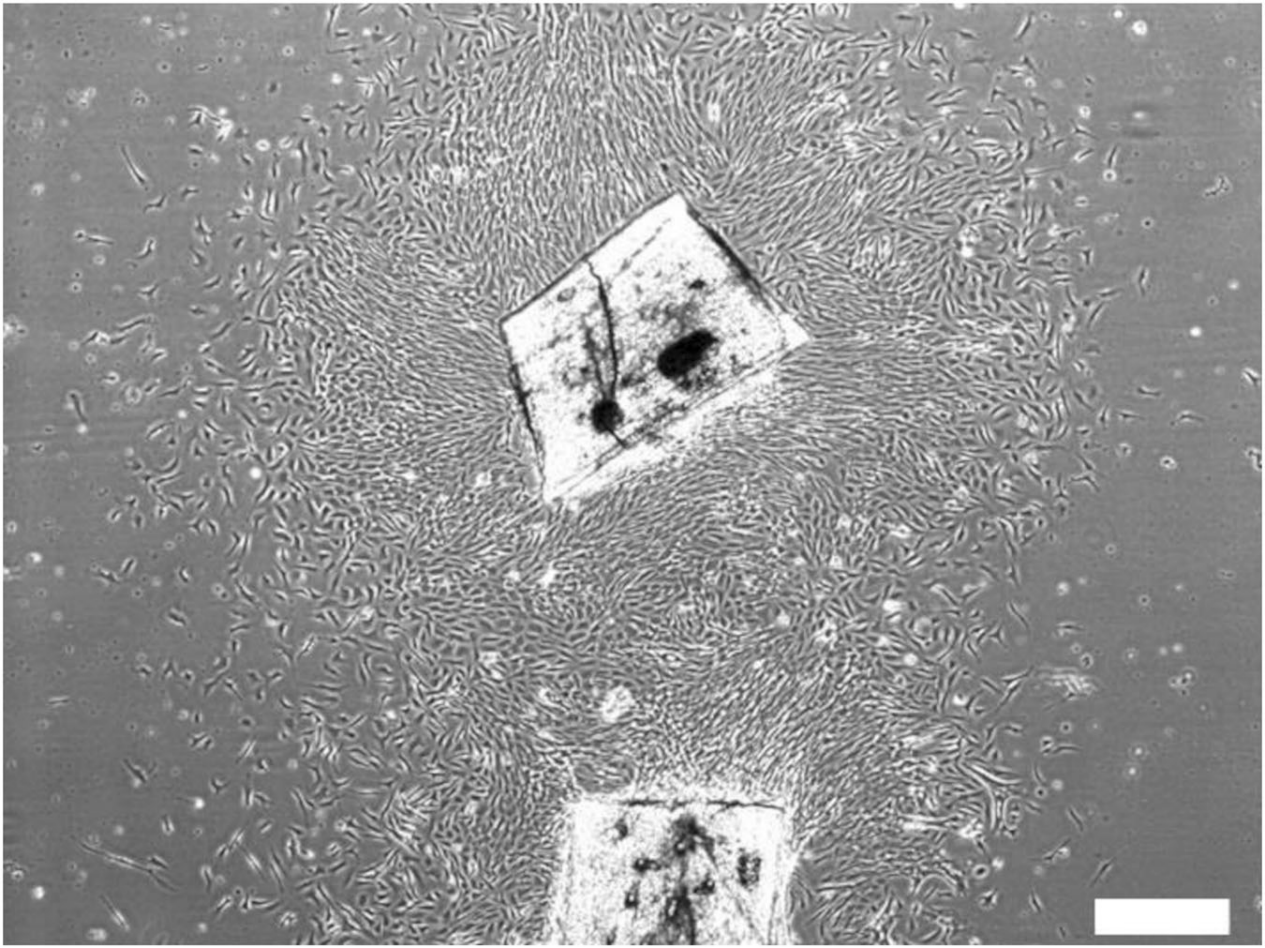
Migratory cells from the fin tissue explants of *S. cirrhifer*. Scale bar: 200 μm.

**Fig. 2:**
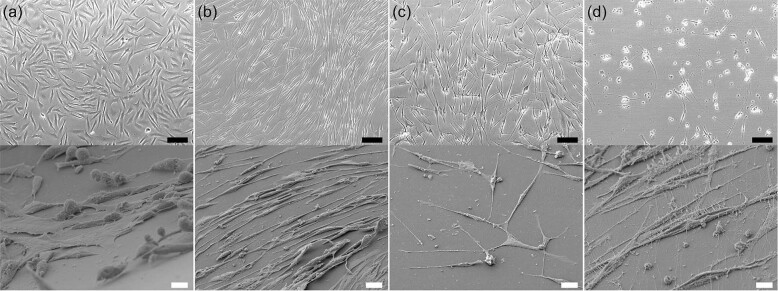
deSc cells differentiated to various cell types. (**a**) Normal fibroblast-like cells, (**b**) skeletal muscle-like cells, (**c**) neural-like cells, (**d**) neurofilaments. Upper: bright field image, scale bar: 50 μm. Lower: SEM image, scale bar: 10 μm.

Cells were obtained from the fin of a thread-sail filefish *Stephanolepis cirrhifer* (*kawahagi* in Japanese name) (Fig. 1). The cell was about 20–50 μm in size. Subculturing was subsequently performed, and stable fibroblast-like cells were obtained at the fifth passage (Fig. 2a, Movie 1). We named the fibroblast-like cells ‘deSc’ (dedifferentiated *Stephanolepis* *cirrhifer*). The deSc cells had been cultured up to 350 passages without CO_2_, and they were immortalized cells. To verify that a chromosomal mutation had not occurred in the deSc cells, Q-banding stain analysis was performed and compared to wildtype *S. cirrhifer*, which had been reported by Murofushi et al.^26^. Thirty-three chromosomes (2n = 30 + X_1_X_2_Y) were found in 96% of the deSc cells, which was identical to the wildtype *S. cirrhifer* (Supplementary Fig. 1). Four percent of the deSc cells showed 66 chromosomes; they might have been in the middle of cell division with replicated DNA.

### Differentiation potency of the fish fibroblast-like cells

To explore the optimal culture conditions for the deSc cells, we examined several culture media, serum and extracellular matrix (ECM), and discovered very interesting characteristics (Table 1). The cells changed their morphology variously depending on the combinations of culture media, serum and ECM (Table 1, Fig. 2b, Movie 2). Of the various results, we focused on the neural-like cells, which differentiated their morphology under fewer culturing factors, that is, without serum (L-15 medium only) in a non-coated flask (Fig. 2c, Movie 3).

**Table 1:** Differentiation of deSc cells under various combinations of culture medium, serum, flask and additive.

Cell Morphology	Culture Medium	Serum	Flask	Additive
Fibroblast-like	L-15	FBS	Collagen I Coated	–
Skeletal muscle-like	AIM V	FBS	Collagen I Coated	–
Neural-like	L-15	–	Non-coated	–
Neurofilament	KBM	FBS	Collagen I Coated	Neural induction supplement
Adipocyte	L-15	SeaGrow	Collagen I Coated	–
Spheroid	L-15	FBS	Spheroid	–
CoCoon	L-15	Horse	Non-coated	–

The cells were cultured at 25 °C, without CO_2_.

Above combinations have been selected as the most effective and are indicated as an example.

**Fig. 3:**
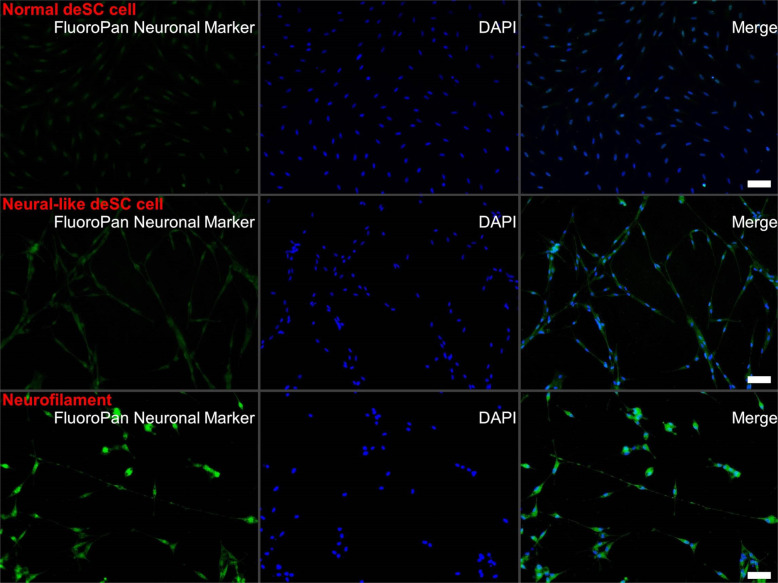
Immunofluorescence of neural deSc cells. Upper: normal deSc cells, middle: neural-like deSc cells, lower: neurofilaments. Scale bar: 50 μm.

The basal state of ES cells in the absence of neural differentiation inhibitors such as serum and transcription factor adopted a neural fate^24^. The deSc cells that were cultured in the same condition also differentiated to neural-like cells within 24 h. This result led us to speculate that the deSc cells had the potential for neural differentiation, likewise if the cells had acquired pluripotency through the dedifferentiation process. To demonstrate this hypothesis, we first attempted to induce neural differentiation directly with KBM Neural Stem Cell medium (Kohjin Bio Co., Ltd.) and Neural Induction Supplement (Thermo Fisher Scientific). The results showed that neurofilaments were formed with a maximum length of 465 μm and an average elongation speed of 45.71 μm/h (Fig. 2d, Movie 4, Table 1). Neural immunofluorescence suggested that the fin cells were virtually differentiated to neural cells (Fig. 3). These results demonstrated that the deSc cells possess the characteristic of practicable direct-differentiation only with culture medium components. We succeeded in inducing neural differentiation, which is the basal state of stem cells, by both the presence/absence of serum and by direct differentiation. We next investigated deSc cell differentiation under stimulation with different sera.

### Cell differentiation with various sera

**Fig. 4:**
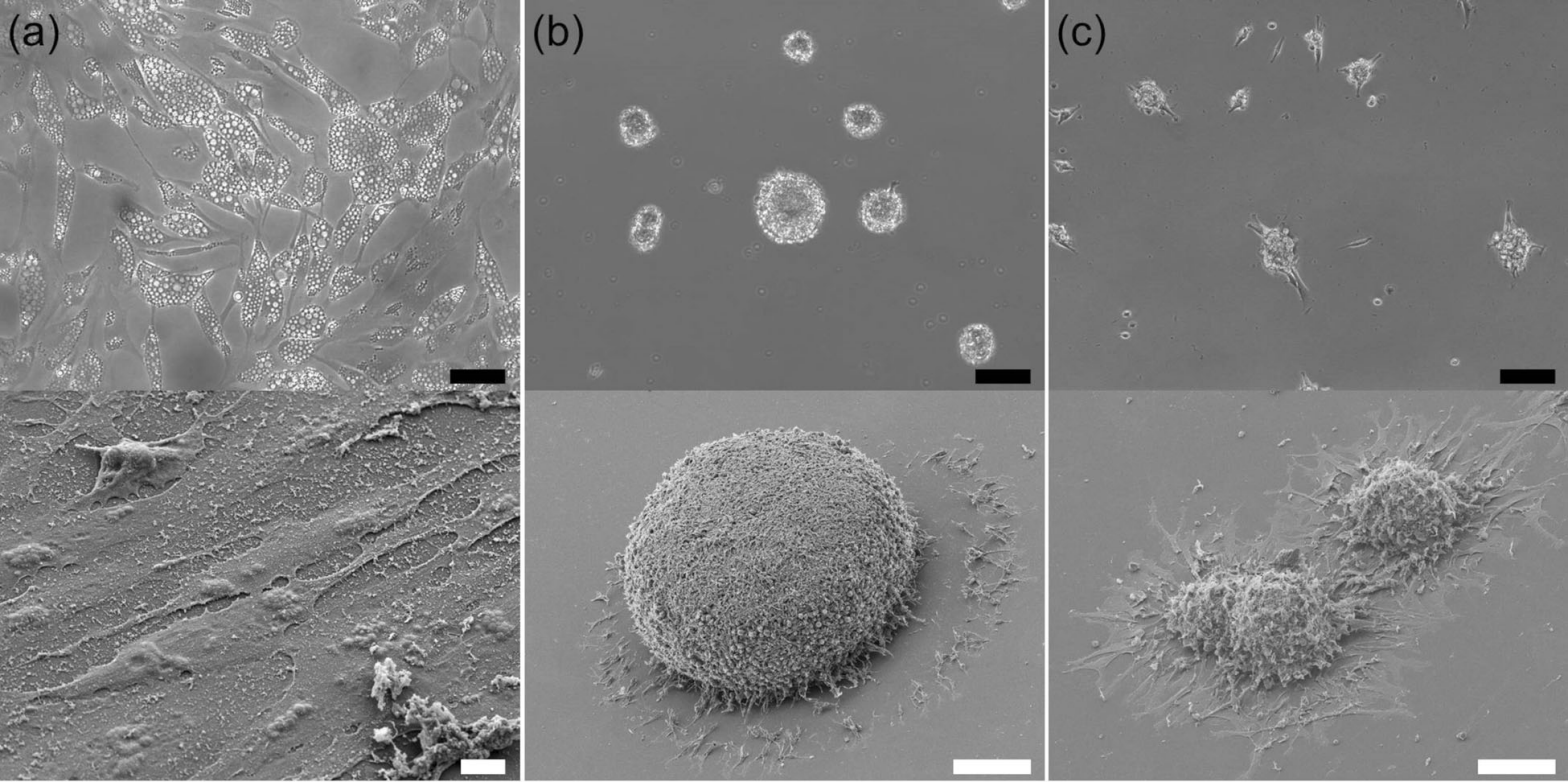
Differentiation of deSc cells to (a) adipocyte, (b) spheroid and (c) CoCoon. Upper: bright field image, scale bar: 50 μm. Lower: SEM image, scale bar: (**a**) 10 μm, (**b**, **c**) 100 μm.

In human iPS cells, culturing with different mammalian sera is reported to affect cell proliferations, differentiations, gene expressions and the stability of transcriptomes^27,28^. We next examined cell differentiation with serum shock. First, we assessed the salmon serum SeaGrow, which was in the same taxonomical group as the deSc cells. Granule-like particles appeared intracellularly in the deSc cells five hours after stimulation with SeaGrow. Another three hours later, the cell morphology became round and larger in size, which were adipocyte-like cells with 0.5–2.0 μm white droplets (Fig. 4a, Movie 5). To examine the white droplets in detail, the cells were stained with Oil Red O and BODIPY, respectively, and also analyzed with gas chromatography. The results showed that the white droplets were fat droplets (Supplementary Fig. 2). Therefore, the use of SeaGrow in the culture media resulted in the differentiation of the deSc cells to adipocytes.

We next examined several mammalian sera other than FBS in the cell culture. With a rabbit and a sheep serum, the deSc cells either did not survive or did not present stable and uniform differentiation states (data not shown).

### Three-dimensional culture aiming at cell molding and design

Organisms consist of various tissues such as bone, cartilage, muscle, and skin, which are established on living scaffolds. At this point, the process of turning differentiated cells into a tissue body was significantly important. A three-dimensional spheroid has been reported as an important in vitro model in terms of similar functions and organization as biological tissues^29^. The deSc cells formed a spheroid with 3D culture, which has been seen in mammalian cells as well (Fig. 4b, Movie 6). Between 20 and 300 μm in size, the spheroids were formed by intaking the surrounding cells. Stimulating the cells with horse serum resulted in colonies that were cell aggregates adherent to the culture flask (Fig. 4c, Movie 7). We named those colonies ‘CoCoon’. The CoCoon migrated in the culture flask at an average speed of 38.46 μm/h and moved largely every 3.5 h by fusing with the surrounding CoCoon (Movie 7). The colonies ranged in size from 20–1000 μm in diameter, and it was possible to macroscopically visualize the largest ones. Moreover, our observations confirmed that the colonies were stable in the culture for at least three weeks as was also seen in the spheroids culture (data not shown). Our results suggested that the fish cells were able to be cultured in 3D both adherently and in suspension.

This 3D culturing and the various cell differentiations, such as spheroids, CoCoon, and skeletal muscle-like cells as well as adipocytes, were reversible processes, except for the neural differentiation. In other words, the differentiated deSc cells reversed their morphology to fibroblast-like cells when the basic culture condition was restored: L-15 medium with 10% FBS in a Collagen I Coated flask (data not shown). From these results, the differentiation processes and the 3D culturing were proven to be simple because the triggers were the culture media, sera and ECM. Moreover, utilising the ease of cell aggregating and processing, we next challenged to create a cultured meat from normal deSc cells. The deSc cell sheet was obtained by continuing to culture the normal deSc cells after they reached their confluent state in a 25–75 cm^2^ cultureware with Collagen I Coated surface to promote cell adhesion and proliferation.

**Fig. 5:**
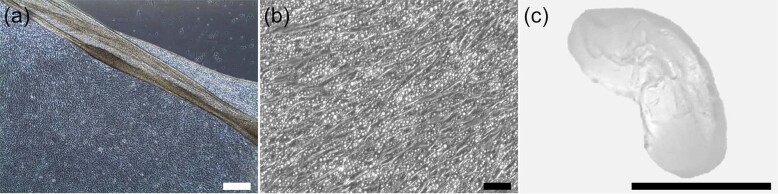
deSc cell sheets and the application. (**a**) Normal cell sheet, (**b**) adipocyte cell sheet, (**c**) prototype of aquatic clean meat shapened with cell sheet technology. Scale bar: (**a**) 200 μm, (**b**) 50 μm, (**c**) 5 cm.

**Fig. 6:**
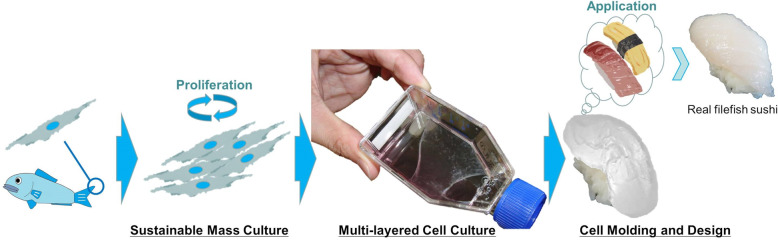
Producing sustainable food: prototype of aquatic clean meat from fish cells. The cell sheet was shapened like sashimi.

deSc cells were able to be cultured and stacked in multiple-layers like a sheet (Fig. 5a). We also succeeded in creating an adipocyte cell sheet under the adipocyte differentiation culturing method (Fig. 5b). Therefore, it was suggested that the differentiation function which had been observed in single deSc cells had not been lost even after the cells formed a sheet structure. Then, the multi-layered deSc cells for aquatic clean meat shrank when the edges of the flask were gently poked with a spatula to detach the cells (Movie 8). The deSc cell sheet was shaped like a fish meat sashimi, producing aquatic clean meat approximately 70 mm long, 30 mm wide, and 2 mm thick at the prototype stage (Fig. 5c). A simple sensory test performed with the artificial sashimi found the following characteristics: 1) the colour was white, 2) there was no smell (no ‘fishy’ smell, which is usually caused by bacteria), 3) there was no taste, 4) the texture was smooth, and 5) the firmness was soft. The shape and size of the aquatic clean meat were flexible. Since it was still very different from real sashimi, it will require further improvement. However, we succeeded in accumulating tiny 20 μm cells to produce edible sashimi at the laboratory level, suggesting that fish cells have the potential to support food sustainability (Fig. 6).

## Discussion

We demonstrated that deSc cell differentiation was regulated by a ‘simple stimulus’ such as medium, serum and ECM without using a specialized technique, such as gene transfer. In general, mammalian cells acquire pluripotency with the introduction of transcription factors^30^. This report does not focus on cell identification, but we confirmed that the deSc cells were positive for alkaline phosphatase (AP), Nanog, Oct4, SSEA-3 and TRA-1-60, respectively (Supplementary Fig. 3). Further examination is required, however, deSc cells may be surprising cells because they may innately possess pluripotency. While the present study focused on the thread-sail filefish *S. cirrhifer*, differentiation phenomena in two dimensions have been observed in 57 kinds of fish cells, including the scorpionfish *Sebastiscus marmoratu*s (Supplementary Fig. 4), with more or less differentiation potential. We named the cells with such inherently pluripotent potential ‘iPX cell’ (inherently Pluripotent X cell), suggesting that these iPX cells may be abundant in fish species. To date, the sophisticated biological technology of gene transfer has enabled the creation of tissues from various kinds of cells, including iPS and ES cells^31^. However, a more efficient and productive technique should be considered for the purpose of food applications^32–34^. Our study showed, for the first time in the world, that fish cells can change themselves dynamically with a simpler treatment than can mammalian cells. This mechanism may be one of the factors that has contributed to diversity in fish. Our results could be an important discovery in clarifying that fish constitute the most diverse group among vertebrates (32,900 fish species)^35^.

We believe that discovering the potential of edible fish to differentiate simply and directly is significant for food sustainability because fish fins are usually discarded as food waste. Our findings could have a large impact on next-generation food processing technologies, including cultured meat. For future studies, the identification and characterization of the deSc differentiated cells will be important in describing the assembled sashimi in detail, which will lead to clarify essential meat components (muscle and adipose cells, muscle proteins and even collagen). By adjusting the ratio of skeletal muscle, fat and neurofilaments in fish cultured meat, the taste and texture of the preferred parts of consumers’ favorite fish can be reproduced in fish meals anytime without reliance on seasonal catches. Moreover, aquatic clean meat has the advantage of not being contaminated by microplastic pollution.

Three important points need to be considered in the development of next-generation foods: 1) Ease of procurement of raw materials or ingredients, 2) ease of processing, and 3) sustainability. With regard to the first point, the raw material for aquatic clean meat is fish fins, which are generally discarded as food waste. In addition, fins can be collected without killing the fish, and the skin that peels off during breeding can be used as a raw material as well. With regard to the second point, easy processing is achievable since our results demonstrated the simple and easy differentiation of fish fin cells into various types of cells, as well as processing that resulted in both layered and 3D cultures of fish cells. Fish clean meat is sustainable and self-sufficient because production can be increased without creating environmental pollution, which is the main advantage of cellular agriculture; this could be a food source in a limited space such as a space shuttle or wherever cells can be cultured. For these three reasons, aquatic clean meat made from fish cells is a strong candidate for a sustainable food resource. In this study, we have found a new way of ‘cooking’, which was not baking and not boiling but, rather, re-cellularizing, that is, obtaining cells from fish fins. A novel biotechnology ‘bio-cooking’ is an interesting future method of food production.

## Materials and methods

### Animal

Live thread-sail filefish *Stephanolepis cirrhifer* was captured near Jogashima Island in Yokosuka City, Kanagawa Prefecture, Japan. Specimens were maintained in aerated artificial seawater, and salinity and water temperature were maintained at 34.0 ppt and 20 ± 2 °C, respectively. All procedures performed in this study involving animals were approved by the Animal Experimental Committee of Japan Agency for Marine-Earth and Technology (JAMSTEC) and conducted in accordance with the Guidelines for the Care of Experimental Animals.

### Preparation of the cell line

The cell line of the thread-sail filefish *S. cirrhifer* was prepared as follows. We obtained a 5 mm^2^ cut of fin tissue from the thread-sail filefish and placed it in 70% ethanol. The tissue was then washed three times with phosphate-buffered saline (PBS), then six times with penicillin and streptomycin (MP Biomedicals, Santa Ana, CA) on ice. The fin tissue was cut into 1 mm squares, placed in 0.25% Trypsin (MP Biomedicals)-0.02% EDTA (MP Biomedicals) solution and incubated at room temperature for 20 min. The tissue was centrifuged at 1100 rpm and washed twice with Leibovitz’s L-15 culture medium (Life Technologies, Carlsbad, CA). The tissue was immersed in L-15 media containing 10% fetal bovine serum (FBS) (Biowest, Nuaillé, France) and 1% Zell Shield (Minerva Biolabs, Berlin, Germany), and then seeded in a 25 cm^2^ Collagen I Coated flask (Thermo Fisher Scientific, Waltham, MA), and cultured in an incubator at 25 °C. After 24 h, we confirmed the outgrowth surrounding the tissue. Several days later, the cells around the tissue were removed with TrypLE Express (Life Technologies), and seeded in a new flask. The culture media was replaced every 3 d and the cells were subcultured at 4.0 × 10^5^ cells/ml five times to establish the cell line, named deSc cells. deSc cells were deposited in the National Institute of Technology and Evaluation (NITE) (Chiba, Japan) with the accession number NITE BP-1369. The cell morphology was observed using an inverted microscope CKX41 (Olympus, Tokyo, Japan) connected to a digital camera ARTCAM-300MI-WOM (Artray, Tokyo, Japan).

### Induction of differentiation

The differentiation of fin cells from the established *S. cirrhifer* cell line was evaluated under various culture conditions. 4.0 × 10^5^ cells were seeded in L-15 media containing 10% FBS in a 25 cm^2^ Collagen I Coated flask or non-coated flask (Thermo Fisher Scientific). Twenty-four hours later, the cells were washed with PBS and treated with different media. Those seeded in a Collagen I Coated flask were treated with AIM V Medium (Thermo Fisher Scientific) + 10% FBS, L-15 + 10% heat-inactivated SeaGrow (EastCoast Biologics, North Berwick, ME) or KBM Neural Stem Cell medium (Kohjin Bio Co. Ltd., Saitama, Japan) + 1X Neural Induction Supplement (Thermo Fisher Scientific). Cells seeded in a non-coated flask were treated with L-15 only. Cell differentiation was photographed every 60 s, and time-lapse movies were created with Axio Vision ver. 4.8 software (Carl Zeiss, Oberkochen, Germany).

### Lipid staining

The fin cells were fixed in 4% paraformaldehyde (Wako Pure Chemical Industries, Ltd., Osaka, Japan) in phosphate-buffered saline (PBS) at RT for 1 h. The fixed cells were subjected to BODIPY staining with an Adipocyte Fluorescent Staining kit (Cosmo Bio Co. Ltd., Tokyo, Japan) following the manufacturer’s protocol. For Oil Red O staining, the fixed cells were treated with 60% isopropanol for 5 min. After the isopropanol was removed, the cells were incubated with Oil Red O solution at RT for 25 min. The treated cells were observed with the microscope as stated above.

### Immunofluorescence

Differentiated cells were fixed in 4% paraformaldehyde (Wako Pure Chemical Industries, Ltd.) in PBS at RT for 12 h. The cells were incubated with a Milli-Mark FluoroPan Neuronal Marker (Mouse IgG conjugated with Alexa 488) (Merck Millipore, Burlington, MA) following the manufacturer’s instructions. The fluorescent cells were observed with a Zeiss Axio Observer. D1 microscope (Carl Zeiss). An AxioCam HRc camera (Carl Zeiss) was used to photograph the images, and the images were analysed using AxioVision ver. 4.8 software (Carl Zeiss).

### Scanning electron microscopy (SEM)

The adherent cells on the Collagen I Coated slide were fixed with 0.4 ml of 2.5% glutaraldehyde in culture medium in a 2 ml culture chamber overnight at 4 °C. The cells were postfixed with 2.0% osmium tetroxide dissolved in PBS for 2 h at 4 °C, and then were dehydrated in a graded series of ethanol and freeze dried in a freeze drier VFD-21S (Vacuum Device Inc., Ibaraki, Japan). They were coated with osmium using a POC-3 osmium plasma coater (Meiwafosis Co. Ltd., Tokyo, Japan) and observed in a field emission scanning electron microscope JSM-6700F (JEOL, Ltd., Tokyo, Japan) operated at 5 kV.

### Gas chromatography

Fatty acids in the fin, the fin cells and the differentiated cells were determined by gas chromatography. The extract sample (1 μl) was injected into a gas chromatograph 7890 A (Agilent Technologies Inc., Santa Clara, CA) equipped with an Omega wax 320 30 m × 0.32 mm.

## Supplementary information

Supplementary Information

Movie 1. Proliferation of deSc cells.

Movie 2. deSc normal cells differentiate to skeletal muscle-like cells.

Movie 3. deSc normal cells differentiate to neural-like cells.

Movie 4. deSc normal cells differentiate to neurofilaments.

Movie 5. deSc normal cells differentiate to adipocyte.

Movie 6. deSc normal cells differentiate to spheroids.

Movie 7. deSc normal cells differentiate to CoCoon.

Movie 8. Shrinking deSc normal cells which were cultured in multiple-layers, scale bar: 1 cm.

## Data Availability

The authors declare that all relevant data supporting the findings of this study are available within the paper and its supplementary information files.
