# Supplementary material for: Reprocessing seafood waste: challenge to develop aquatic clean meat from fish cells
Source: NPJ Sci Food. 2022 Jan 27;6:7. doi: 10.1038/s41538-021-00121-3 (PMC8795430; doi:10.1038/s41538-021-00121-3)
Supplement: Supplementary file 1 — Supplementary Information [file 41538_2021_121_MOESM1_ESM.pdf]

Supplementary Information

Reprocessing seafood waste:
Challenge to develop aquatic clean meat from fish cells

Yusuke Tsuruwaka[1,2,3]*, Eriko Shimada[1,4,5]

[1]Cellevolt, Niigata, Japan

[2]Institute for Advanced Biosciences, Keio University, Yamagata, Japan

[3]Marine Bioresource Exploration Research Team, Marine Biodiversity Research Program, Institute of Biogeosciences, Japan Agency for Marine-Earth Science and Technology (JAMSTEC), Kanagawa, Japan

[4]Division of Applied Biosciences, Graduate School of Agriculture, Kyoto University, Kyoto, Japan

[5]Department of Pharmacology, University of California, Davis, Davis, California, USA

***Corresponding author:**
Yusuke Tsuruwaka
Email: tsuru@ttck.keio.ac.jp
Email: ytsuruwaka@gmail.com

Co-author:
Eriko Shimada
Email: eshimada@alumni.ucdavis.edu

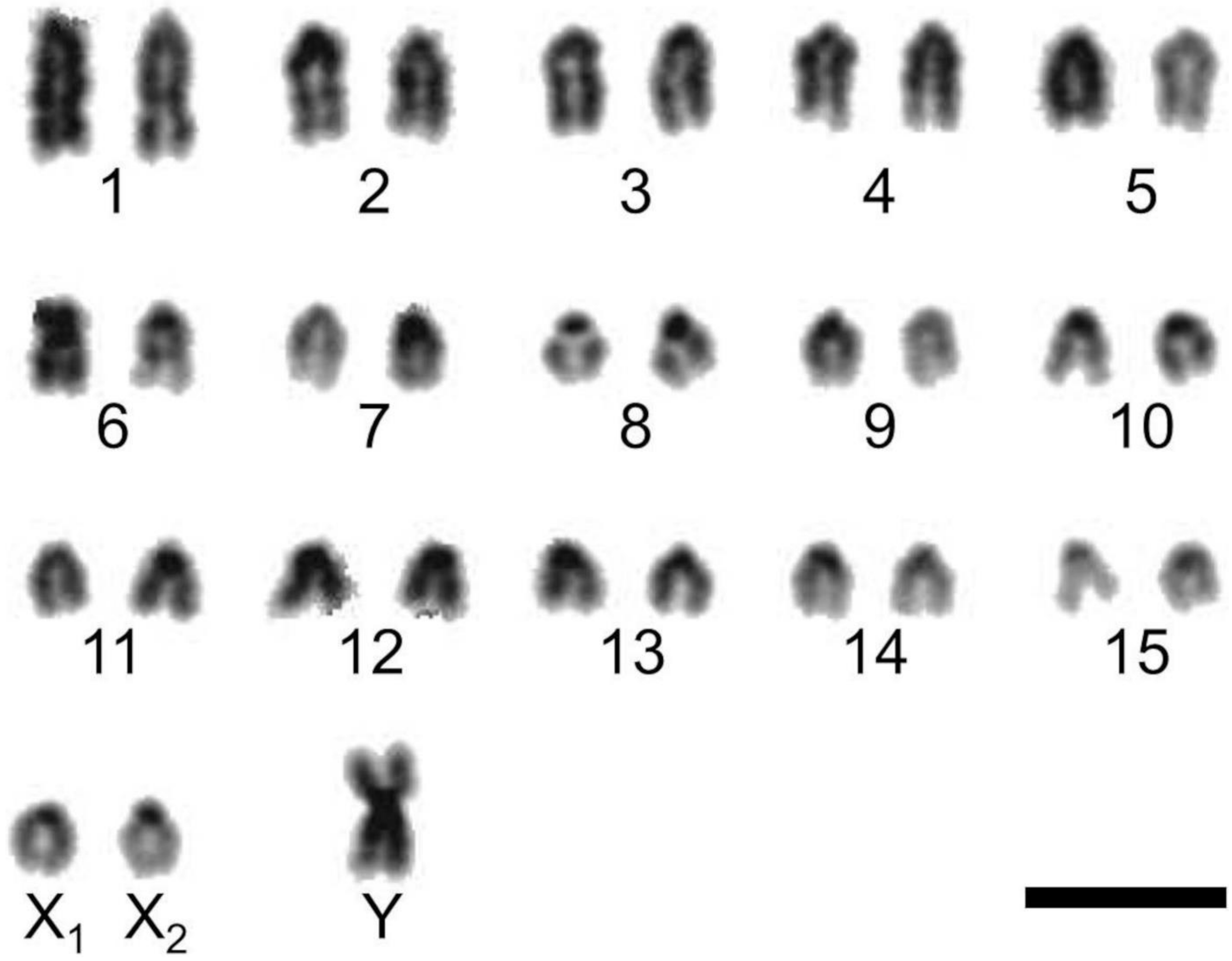

Supplementary Figure 1. Chromosome of deSc cells, *Stephanolepis cirrhifer*, 2n=30+X_1X_2Y(33). Scale bar: 5 μm. Q-banding stain analysis was performed according to Murofushi et al.[1].

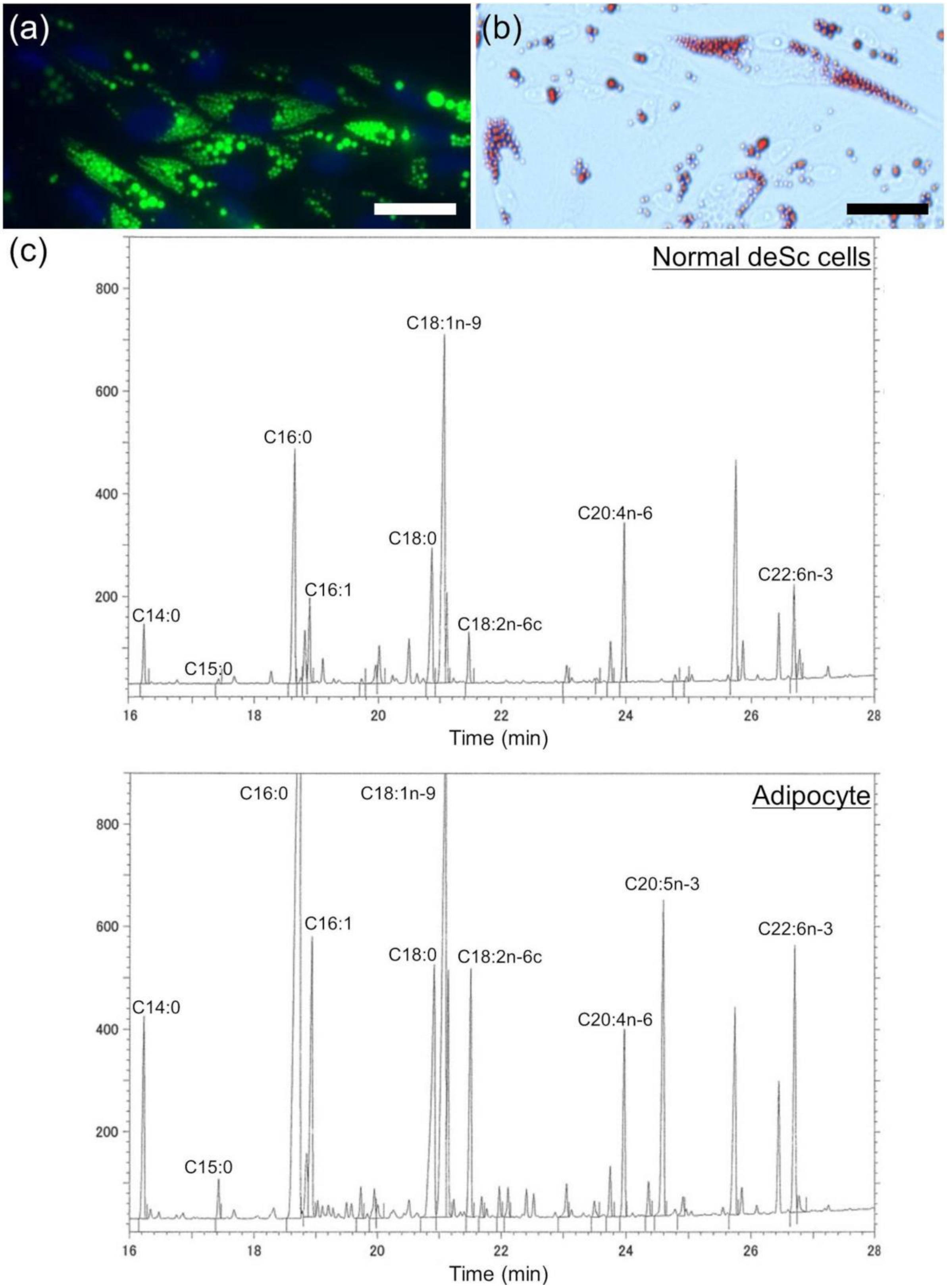

Supplementary Figure 2. Adipocyte staining with (**a**) BODIPY and (**b**) Oil Red O. Scale bar: 50 μm. (**c**) Gas chromatogram of normal deSc cells (upper) and adipocyte (lower).

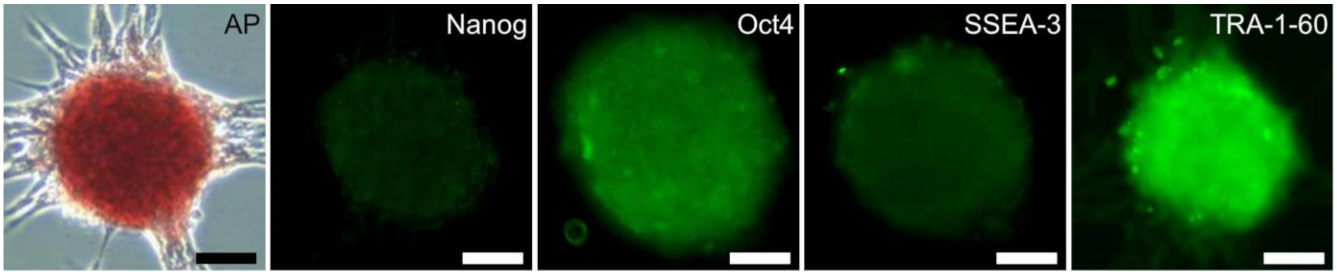

Supplementary Figure 3. CoCoon staining with alkaline phosphatase (AP), Nanog, Oct4, SSEA-3, and TRA-1-60 straining kit (Human ES/iPS cell Characterization Kits, System Biosciences). Scale bar: 100 μm.

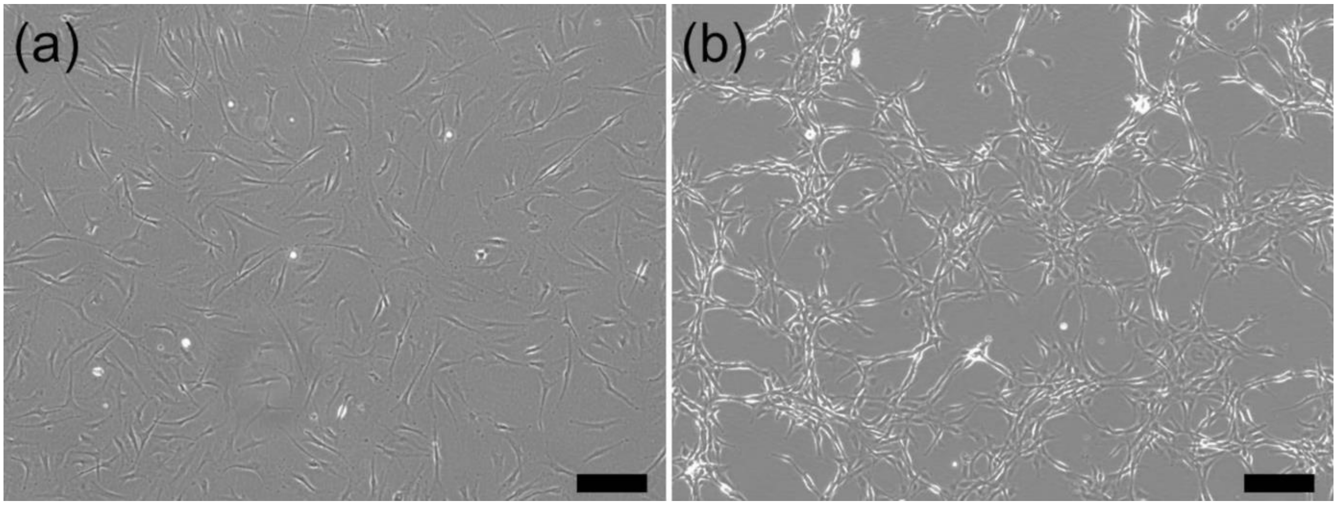

Supplementary Figure 4. Differentiation of scorpion fish, *Sebastiscus marmoratu*s. (**a**) normal, (**b**) neural-like cells under bright field. Scale bar: 50 μm.

Supplementary Reference

1. Murofushi, M., Oikawa, S., Nishikawa, S. & Yosida, T. H. Cytogenetical studies on fishes, III. Multiple sex chromosome mechanism in the filefish, *Stephanolepis cirrhifer*. *Japan. J. Genetics* **55**, 127–132 (1980).

Supplementary Movie

Movie 1. Proliferation of deSc cells.

Movie 2. deSc normal cells differentiate to skeletal muscle-like cells.

Movie 3. deSc normal cells differentiate to neural-like cells.

Movie 4. deSc normal cells differentiate to neurofilaments.

Movie 5. deSc normal cells differentiate to adipocyte.

Movie 6. deSc normal cells differentiate to spheroids.

Movie 7. deSc normal cells differentiate to CoCoon.

Movie 8. Shrinking deSc normal cells which were cultured in multiple-layers. Leica L2 microscope equipped with a CoolSNAP cooled 5.0 CCD camera was used to shoot. Scale bar: 1 cm.
